# Supplementary material for: Alpha-Emitter Radiopharmaceuticals and External Beam Radiotherapy: A Radiobiological Model for the Combined Treatment
Source: Cancers (Basel). 2022 Feb 21;14(4):1077. doi: 10.3390/cancers14041077 (PMC8870677; doi:10.3390/cancers14041077)
Supplement: Supplementary file 1 [file cancers-14-01077-s001.zip › cancers-1565763-supplementary.pdf]

cancers MDPI

Supplementary Material S1

Embase Session Results

Query

('bone metastasis'/exp OR 'bone invasion' OR 'bone metastasis' OR 'bone metastatic tumor' OR 'bone metastatic tumour' OR 'bone tumor, metastatic' OR 'bone tumour, metastatic' OR 'ilium metastasis' OR 'metastasis, bone' OR 'metastasis, ilium' OR 'metastatic bone tumor' OR 'metastatic bone tumour' OR 'osseous metastasis' OR 'osteoblastic metastasis' OR 'osteoplastic metastasis' OR 'skeletal metastasis' OR 'skeleton metastasis' OR 'skull metastasis')

AND ('radium chloride ra 223'/exp OR 'alpharadin' OR 'bay 88 8223' OR 'bay 88-8223' OR 'bay88 8223' OR 'bay88-8223' OR 'radium 223 chloride' OR 'radium 223 dichloride' OR 'radium chloride ra 223' OR 'radium dichloride ra 223' OR 'radium ra 223 chloride' OR 'radium ra 223 dichloride' OR 'radium ra-223 dichloride' OR 'radium ra223 dichloride' OR 'xofigo')

AND ('external beam radiotherapy'/exp OR 'ebrt (external beam radiation therapy)' OR 'ebrt therapy' OR 'external beam rt' OR 'external beam irradiation' OR 'external beam radiation' OR 'external beam radiation therapy' OR 'external beam radiotherapy' OR 'external beam radiotherapy' OR 'external beam therapy' OR 'external radiotherapy')

AND ('toxicity'/exp OR 'hypertoxicity' OR 'subacute toxicity' OR 'tissue toxicity' OR 'toxic actions' OR 'toxic effect' OR 'toxicity' OR 'toxigenicity' OR 'neutropenia'/exp OR 'cyclic neutropaenia' OR 'cyclic neutropenia' OR 'neutropaenia' OR 'neutropenia' OR 'overall survival'/exp OR 'overall survival')

AND ('randomized controlled trial'/exp OR 'controlled trial, randomized' OR 'randomised controlled study' OR 'randomised controlled trial' OR 'randomized controlled study' OR 'randomized controlled trial' OR 'trial, randomized controlled' OR 'clinical trial'/exp OR 'clinical drug trial' OR 'clinical trial' OR 'major clinical trial' OR 'trial, clinical')

AND (2010:py OR 2011:py OR 2012:py OR 2013:py OR 2014:py OR 2015:py OR 2016:py OR 2017:py OR 2018:py OR 2019:py OR 2020:py)

AND 'Article'/it

Results

25

Date

23 April 2021

Supplementary Material S2

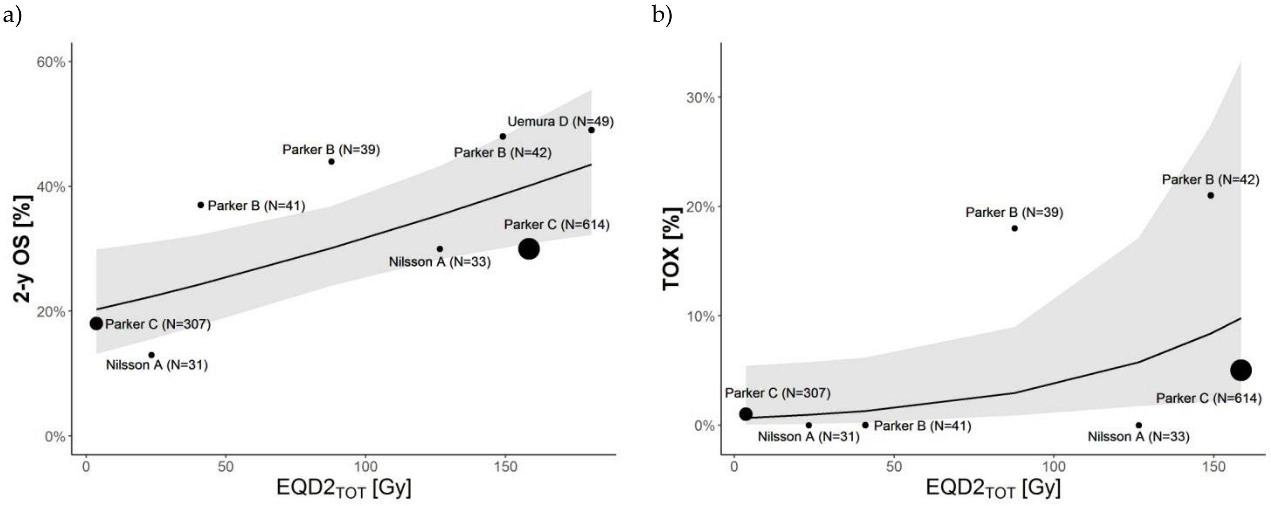

Figure S1. Logistic regression model for a) two years overall survival (2-y OS) and b) toxicity impact (TOX) measured as neutropenia rate. Predicted values are reported at study-level (fixed-effect only). The dots' dimensions are proportional to the number of patients included in the study (Table 2). The grey area represents the 95% CI. Models' parameters are reported in Table S1. Note that study A (Nilsson et al [8]) with numerosity of 31 and study C (Parker et al [7]) with numerosity of 307 are both ^{223}Ra placebo studies. The EBRT schedule considered is equal to 20 Gy delivered in 5 fractions.

Table S1. Models' parameters of logistic regression for both 2-y OS and TOX impact measured as neutropenia rate, assuming the EBRT schedule of 20 Gy in 5 fractions.

	**2-y OS model**			
	**β**	**SE**	**z statistic**	**p-value**
Intercept	-1.390	0.269	-5.176	<0.001
EQD2TOT	0.006	0.002	2.741	0.006
	**TOX model**			
	**β**	**SE**	**z statistic**	**p-value**
Intercept	-5.076	1.126	-4.509	<0.001
EQD2TOT	0.018	0.009	1.931	0.05

EQD2TOT is obtained considering the ^{223}RaCl2 administration of reported schedules + EBRT (8 Gy in 1fr). β indicates the model parameters of intercept and slope. SE = standard error.

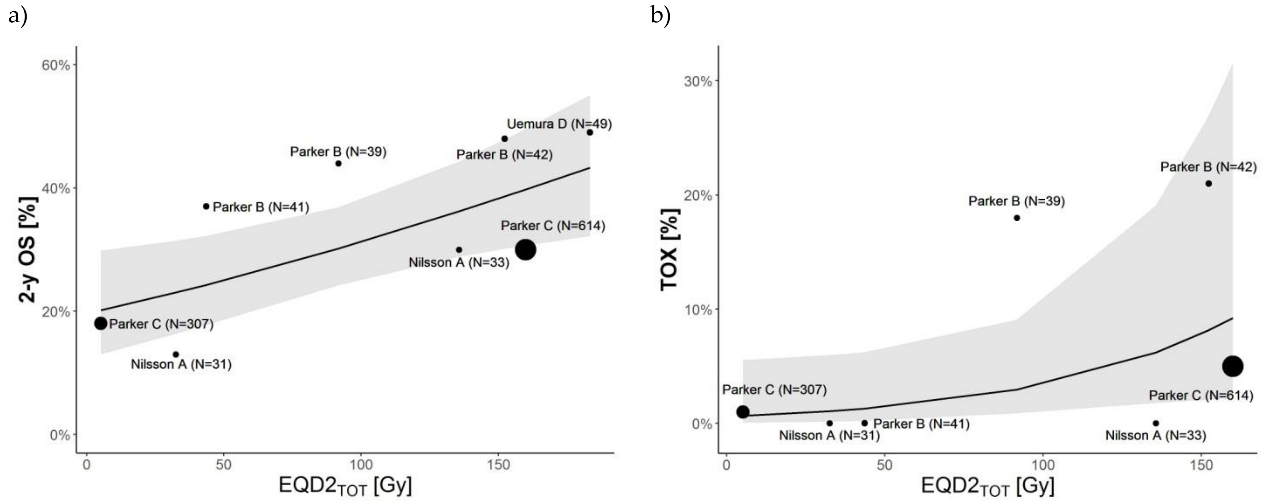

Figure S2. Logistic regression model for a) two years overall survival (2-y OS) and b) toxicity impact (TOX) measured as neutropenia rate. Predicted values are reported at study-level (fixed-effect only). The dots' dimensions are proportional to the number of patients included in the study (Table 2). The grey area represents the 95% CI. Models' parameters are reported in Table S2. Note that study A (Nilsson et al [8]) with numerosity of 31 and study C (Parker et al [7]) with numerosity of 307 are both ^{223}Ra placebo studies. The EBRT schedule considered is equal to 20 Gy delivered in 5 fractions.

Table S2. Models' parameters of logistic regression for both 2-y OS and TOX impact measured as neutropenia rate, assuming the EBRT schedule of 30 Gy in 10 fractions.

	**2-y OS model**			
	**β**	**SE**	**z statistic**	**p-value**
Intercept	-1.408	0.274	-5.138	<0.001
EQD2TOT	0.006	0.002	2.736	0.006
	**TOX model**			
	**β**	**SE**	**z statistic**	**p-value**
Intercept	-5.101	1.148	-4.444	<0.001
EQD2TOT	0.018	0.009	1.895	0.06

EQD2TOT is obtained considering the $^{223}RaCl2$ administration of reported schedules + EBRT (8 Gy in 1fr). β indicates the model parameters of intercept and slope. SE = standard error.
